# A Comparison of Multiple Methods for Estimating Parasitemia of Hemogregarine Hemoparasites (Apicomplexa: Adeleorina) and Its Application for Studying Infection in Natural Populations

**DOI:** 10.1371/journal.pone.0095010

**Published:** 2014-04-17

**Authors:** João P. Maia, D. James Harris, Salvador Carranza, Elena Gómez-Díaz

**Affiliations:** 1 CIBIO Research Centre in Biodiversity and Genetic Resources, InBIO, Universidade do Porto, Campus Agrário de Vairão, Vairão, Vila do Conde, Portugal; 2 Departamento de Biologia, Faculdade de Ciências, Universidade do Porto, Porto, Portugal; 3 Institut de Biologia Evolutiva (CSIC-Universitat Pompeu Fabra), Barcelona, Spain; Universidade Federal de Minas Gerais, Brazil

## Abstract

Identifying factors influencing infection patterns among hosts is critical for our understanding of the evolution and impact of parasitism in natural populations. However, the correct estimation of infection parameters depends on the performance of detection and quantification methods. In this study, we designed a quantitative PCR (qPCR) assay targeting the 18 S rRNA gene to estimate prevalence and intensity of *Hepatozoon* infection and compared its performance with microscopy and PCR. Using qPCR, we also compared various protocols that differ in the biological source and the extraction methods. Our results show that the qPCR approach on DNA extracted from blood samples, regardless of the extraction protocol, provided the most sensitive estimates of *Hepatozoon* infection parameters; while allowed us to differentiate between mixed infections of Adeleorinid (*Hepatozoon*) and Eimeriorinid (*Schellackia* and *Lankesterella*), based on the analysis of melting curves. We also show that tissue and saline methods can be used as low-cost alternatives in parasitological studies. The next step was to test our qPCR assay in a biological context, and for this purpose we investigated infection patterns between two sympatric lacertid species, which are naturally infected with apicomplexan hemoparasites, such as the genera *Schellackia* (Eimeriorina) and *Hepatozoon* (Adeleorina). From a biological standpoint, we found a positive correlation between *Hepatozoon* intensity of infection and host body size within each host species, being significantly higher in males, and higher in the smaller sized host species. These variations can be associated with a number of host intrinsic factors, like hormonal and immunological traits, that require further investigation. Our findings are relevant as they pinpoint the importance of accounting for methodological issues to better estimate infection in parasitological studies, and illustrate how between-host factors can influence parasite distributions in sympatric natural populations.

## Introduction

Parasites can be major drivers of host ecology and evolution and play key roles in ecosystem functioning and structure. Parasitism can for example alter host life-history traits and fitness [1], [2], as well as influence predator-prey or competitive interactions between and within species [3]–[5]. However, the ecological niche in which both host and parasite occur is complex and multifaceted, and there is considerably variation in patterns of parasitism in natural populations at multiple spatial and temporal scales [6], from individual to community levels [7], [8]. Natural variation in infection parameters can be due to differences in the environmental exposure to parasites [9], or to variation in the susceptibility or resistance to infection [10]. Investigating the underlying patterns can contribute to a better understanding of the impact and evolution of parasitism [11]–[13], while also providing valuable epidemiological and conservation information.

Importantly, the estimation of biological relevant and realistic infection patterns is constrained by differences in the accuracy, specificity and sensitivity of the detection and quantification protocols used [14], [15]. The use of less accurate protocols may lead to erroneous ecological and epidemiological inferences, which is particularly critical in host-parasite systems with low intensity levels or with mixed parasite infections. In recent years, quantitative PCR (qPCR) has been used in parasitological studies as it allows the simultaneous detection and quantification of parasite DNA from various biological sources, such as host and vector blood and tissues [16]–[20]. Compared to more traditional approaches, such as microscopy or conventional PCR (PCR), qPCR has increased accuracy and sensitivity of detection [21], [22]. Despite the advantages, the use of this quantitative method in parasitology has been primarily used for clinical samples and/or pathogens of human-health and veterinary importance [17]. Although this technique is now routinely applied for clinical studies in domestic animals [21], its applications in wild animal populations, is just beginning to emerge [6], [19], [23]–[25]. More importantly, studies comparing the performance of the different methods under various experimental conditions are generally lacking (but see [15]).

Sympatric and closely related species represent natural model systems and an ideal opportunity where to investigate between-host differences in infection parameters [26]. Closely related species are likely to be susceptible to the same or similar parasite species due to common ancestry, and the same can happen with sympatric host species due to common ecological determinants [27]. In these systems, differences in infection may arise due to inter- and intra-host variation in susceptibility and/or tolerance to infection, while controlling for general environmental differences in exposure. Reptiles have become model organisms for parasitological studies, in part due to their low dispersal potential, and the occurrence of sympatric speciation [28]. Despite the growing number of studies addressing infection variation in animal populations [29]–[32], these remain largely enigmatic in the case of wild reptiles. In reptiles, body size and weight have been commonly used as a proxy measure to explain individual as well as population and species differences in susceptibility and/or tolerance to parasite infection [33]–[37], because it is often positively correlated with host life-history traits such as longevity, survival and fecundity, which relate to fitness [38]. Other factors that might drive variation in parasite infection include host behaviour and morphology [39], [40], immunity [10], [41] and ecology [27], [42]. However, previous studies have shown contrasting patterns of association with those factors [34]–[36], [43], which may be in part due to the effects of sampling and methodological bias, and/or confounding phylogenetic or environmental factors [42], [44], [45].

*Hepatozoon* (Apicomplexa: Adeleorina), is a highly diverse genus of intracellular hemogregarine parasites with more than 300 species described [46], and the most common and widely distributed hemoparasites found in reptiles [47]. Yet few studies to date have examined prevalence and intensity of infection in wild reptile populations (but see [35], [37], [48]–[51]). Arthropod vectors, such as mites, ticks and mosquitoes, are the definitive hosts in a complex heteroxenous lifecycle that includes a wide range of vertebrates as intermediate hosts [46]. Pathogenesis caused by *Hepatozoon* infections in reptiles is unclear, with studies reporting from apparently non-detrimental infections in natural hosts [36], [52], to severe and life-threatening illness in unnatural hosts [53]. This has been mostly studied in domestic animals, for which the most common symptoms include anaemia, lethargy, weight loss, weakness and cachexia [54], [55], while the pathogenic effects on wildlife are mostly unknown. In addition, other apicomplexans, such as *Schellackia* sp. (Apicomplexa: Eimeriorina), are often found at lower prevalence and intensities in wild reptile hosts, and mixed infections with *Hepatozoon* can also occur [56], [57]. In this study, we investigate *Hepatozoon* infection parameters in two sympatric closely related lacertid species, *Podarcis bocagei* and *P. hispanica*. These two host species present an attractive system because: first, they live in sympatry and, despite having similar ecological requirements, there are apparent preferences for microhabitat: *P. hispanica* is frequently found on rocks (saxicolous), while *P. bocagei* is mainly ground­dwelling [58], [59]; second, despite being genetically closely related [60], they present considerable morphological differences, with *P. bocagei* being generally larger [61], [62]; third, size sexual dimorphism has been reported for both species, with adult males being larger than females [62], [63]; and fourth, recent studies report high prevalence of *Hepatozoon* and low prevalence of *Schellackia* infections for these species in the Iberian Peninsula [50], [56]. Therefore, this model system provides an ideal scenario for investigating between host-species, sex and inter-individual differences in patterns of *Hepatozoon* infection, while controlling for confounding factors such as ecology and host and parasite phylogeny.

The objectives of this study are: i) to evaluate the accuracy and sensitivity of different detection methods (microscopy, PCR and qPCR) and protocols (biological source and DNA extraction protocol) for detecting and quantifying hemogregarine parasites in reptile samples, as well as the occurrence of mixed infections, and ii) apply the most sensitive approach, i.e. qPCR, to determine prevalence and intensity of *Hepatozoon* infection in two sympatric closely related lacertid species, *P. bocagei* and *P. hispanica*, and assess the relative role of inter-individual (intra-species) and between-species factors on these parameters.

## Materials and Methods

### Study species and study site

Samples were collected from two lacertid lizard species, *P. bocagei* and *P. hispanica*, from a single location in Gerês, Northern Portugal (41.782340, -8.145140), in July 2011. Capture permits were issued by the ICNB (Instituto da Conservação da Natureza e da Biodiversidade, I.P.), license numbers 67-75/2011/CAPT. These hosts are small, diurnal, insectivorous lizards, with adult snout-vent length (SVL) of 45–65 mm and 37–70, respectively. A total of 87 adult individuals were sampled (51 *P. bocagei* and 36 *P. hispanica*, see Table 1). Each individual was handled by experienced herpetologists, a small piece of the tail-tip was collected and, when enough blood was obtained, a blood drop was stored in Whatman filter paper and the rest was used to make a blood smear. No animals were sacrificed. SVL was measured using a vernier caliper, animals were photographed and sex was determined based on the existence of enlarged femoral pores. After processing, animals were released at the site of capture. This protocol has been approved by the ethical committee of the University of Porto. Tissue samples were preserved in 96% ethanol and stored at room temperature and blood drops stored at −20°C. Blood smears were air-dried, fixed with methanol on the day of collection and stained with diluted Giemsa (1∶9 of distilled water) for 55 minutes within a week of collection.

**Table 1:** Prevalence and mean intensity of *Hepatozoon* and Eimeriorina parasites for the two lizard species analysed in this study, estimated using three different methods.

		*Hepatozoon*					Eimeriorina			
		qPCR		PCR	Microscopy		qPCR (Blood Kit)	PCR	Microscopy*	
Host species	Sex	Prevalence	Mean Intensity (log(copy number))	Prevalence	Prevalence	Mean Intensity (% ± std)	Prevalence	Prevalence	Prevalence	Mean Intensity (% ± std)
*Podarcis*	Female	13/22 (59%)	1.23±0.26	10/22 (45%)	5/16 (31%)	0.12±0.10	0/22 (0%)	1/22 (5%)	0/16 (0%)	-
*bocagei*	Male	24/29 (83%	2.20±0.28	17/29 (59%)	14/25 (56%)	0.22±0.08	3/29 (10%)	2/29 (7%)	3/25 (12%)	0.16±0.12
		37/51 (73%)	1.74±0.20	27/51 (53%)	19/41 (46%)	0.18±0.06	3/51 (6%)	3/51 (6%)	3/41 (7%)	-
*Podarcis*	Female	16/18 (89%)	1.80±0.26	11/18 (61%)	7/14 (50%)	0.11±0.05	0/18 (0%)	0/18 (0%)	1/14 (7%)	0.02
*hispanica*	Male	16/18 (89%)	2.94±0.33	13/18 (72%)	12/17 (71%)	0.73±0.24	1/18 (6%)	2/18 (11%)	1/17 (6%)	0.02
		32/36 (89%)	2.37±0.23	24/36 (67%)	19/31 (61%)	0.45±0.15	1/36 (3%)	2/36 (6%)	2/31 (6%)	0.02±0.00
		*n* = 87 (79%)	*n* = 81	*n* = 87 (59%)	*n* = 72 (53%)	*n* = 72	*n* = 87 (5%)	*n* = 87 (6%)	*n* = 72 (7%)	*n* = 72

**Figure 1:**
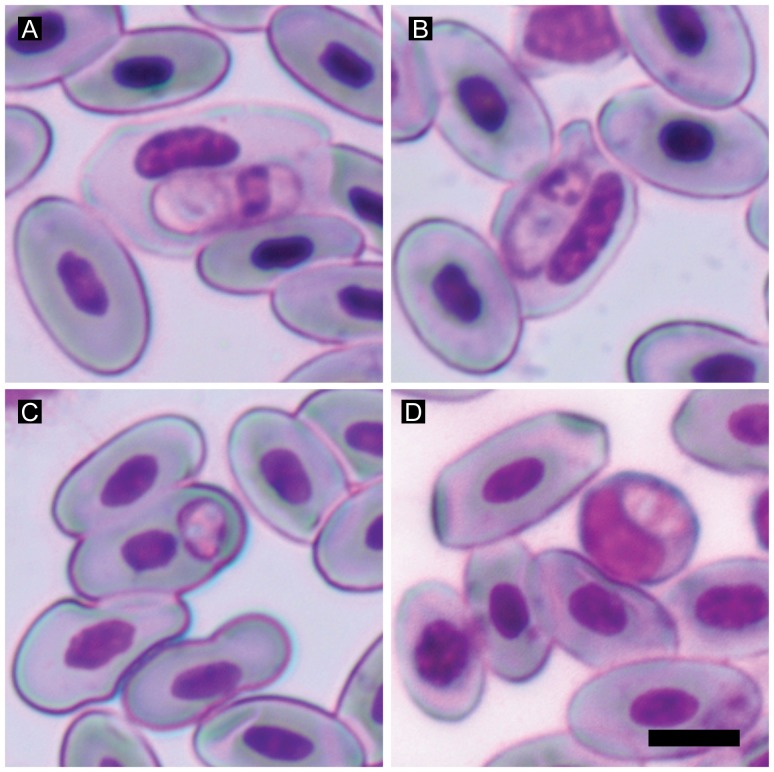
Parasites found in common wall lizards analysed from Gerês, Portugal. *Hepatozoon* sp. infecting erythrocytes from *P. bocagei* (A), and *P. hispanica* (B). *Schellackia* sp. infecting an erythrocyte from *P. bocagei* (C), and a leukocyte from *P. hispanica* (D). Scale bar = 5 µm.

* These were found inside erythrocytes, except for one that was found in a leukocyte (Fig. 1).

### Microscopic examination

Blood smears were examined using an Olympus CX41 microscope with an in-built digital camera (SC30). Several photographs per slide were taken under the 40× magnification lenses and stitched using cell∧B software (basic image-acquisition and archiving software). Prevalence was estimated as the proportion of infected hosts and intensity of infection was estimated as the number of parasites per 4,000 erythrocytes from a total of 72 blood smears [64], [65]. Counts were done using the manual cell counter plug-in available in the image processing software ImageJ ver. 1.44p [66]. Fig. 1 shows mature intraerythrocytic gamonts of *Hepatozoon* sp. and sporozoites of *Schellackia* sp. infecting the two lizard species.

### DNA extraction and sample preparation

For the total number of samples (*n* = 87), DNA was extracted from blood using the Speedtools tissue DNA extraction kit (Biotools, Madrid). In a subset of 47 individuals, in which we compared various methodological approaches, DNA was extracted from two biological sources: blood drops stored in Whatman filter paper (approximately 3 mm by 3 mm) and tail-tip muscle tissue (approximately 2 mm by 2 mm with skin removed). On these samples we used two extraction protocols: the Speedtools commercial kit following manufacturer's instructions, and the standard saline protocol [67]. Briefly, the saline method used consisted of adding 600 µl of lysis buffer (0.5 M tris; 0.1 M EDTA; 2% SDS; pH 8.0) and 10 µl of proteinase K (25 mg/ml) to the cut material, which was incubated at 56°C overnight. After incubation, 300 µl of ammonium acetate (5 M; pH 8.0) was added and centrifuged for 15 min at 14000 rpm at 4°C. The supernatant was carefully transferred to new eppendorf tubes, 600 µl of ice-cold isopropanol was added and samples were frozen between 3 h to overnight. After incubation, samples were centrifuged for 25 min at 14000 rpm at 4°C, and the supernatant was carefully discarded. Then, 1000 µl of ice-cold ethanol (70%) was added and centrifuged for 15 min at 14000 rpm at 4°C. The supernatant was carefully discarded and samples were left to evaporate at room temperature. When completely evaporated, 50 µl of ultra-pure water was added and samples were left to hydrate at ambient temperature in an agitator for 2 h. For qPCR analyses, samples were diluted to 10 ng/µl with nuclease-free water (QIAGEN) using an ND-1000 Spectrophotometer (NanoDrop Technologies, Inc.). Quality of DNA for all samples was verified with the nm wavelength within the excepted range for pure DNA according to the manufacturer's manual (260 nm/280 nm ratio mean was 1.98, and the 260 nm/230 nm ratio mean was 2.1). This standardization procedure allows to control for the amount of host DNA, which can interfere with amplification of parasite DNA [16], [68].

### Conventional PCR, sequencing and phylogenetic analysis

PCR amplifications were performed on all 87 samples using primers HepF300 and HepR900 [69] that target part of the *Hepatozoon* 18 S rRNA gene. The PCR reactions using the Hep primers were run in a 20 µl reaction mixture containing 1 U of GoTaq DNA Polymerase (5 u/µl), 1.5 mM MgCl_2_ (25 mM), 0.125 mM of each nucleotide, 1 X GoTaq Flexi Buffer, 0.6 mM of each primer, and 2 µl of DNA. The reaction mix was heated to 94°C for 3 min, and amplification was performed through at 94°C for 30 s, 60°C for 30 s, and 72°C for 1 min, in 35 cycles, with a final 10 min extension at 72°C. Two negatives (one blank and one known *Hepatozoon* negative) and one positive control (one known *Hepatozoon* positive) were run with each reaction. Sequencing of the positive PCR products was performed in both directions outsource (Macrogen Europe, The Netherlands). Geneious v6.0.3 was used for contig assembly and visualization of sequences. The “heterozygotes plugin” with 30% peak similarity was used to search for possible heterozygous positions. Consensus sequences for each individual were deposited in GenBank under the accession numbers KJ189387-KJ189433 (*Hepatozoon*), KJ189382-KJ189385 (*Schellackia*) and KJ189386 (*Lankesterella*). One representative of each of the three *Hepatozoon* haplotypes (KJ189418, KJ189426 and KJ189390), of the two *Schellackia* haplotypes (KJ189382 and KJ189384), and the single *Lankesterella* haplotype (KJ189386), were aligned with published data on GenBank using the MUSCLE alignment [70] with default parameters and “Refine Existing Alignment” option implemented in Geneious. The alignment consisted of 75 taxa and was 641 bp long. Two different phylogenetic analyses (Maximum Likelihood and Bayesian Inference) were conducted. Maximum Likelihood (ML) analysis with random sequence addition (100 replicate heuristic searches) was used to assess evolutionary relationships, using the software PhyML 3.0 [71]. Support for nodes was estimated using the bootstrap technique [72] with 1000 replicates. The AIC criterion conducted in jModeltest 0.1.1 [73] was used to choose the model of evolution (TIM1+G). Bayesian analysis was implemented using Mr. Bayes v.3.1 [74] with parameters estimated as part of the analysis. The analysis was run for 10×10^6^ generations, saving one tree with random tree sampling each 1000 generations. All trees prior to reaching stationarity (25% of the run) were discarded as burn-in samples, and the remaining trees were combined in a 50% majority consensus tree.

### qPCR design and protocol

qPCR assays for detecting *Hepatozoon* species in canids have been previously designed [18], [20]. We first tested the former protocol in reptile-infected samples, and we obtained a double dissociation peak in the Melting Curve Analysis (MCA). Therefore we designed a qPCR assay targeting the 18 S rRNA gene of hemogregarine species in reptiles taking into account the variation previously reported in *Hepatozoon* and other apicomplexans (Fig. S1). For this purpose we designed Hemogregarine specific primers JM4_F (5′-ACTCACCAGGTCCAGACATAGA-3′) and JM5_R (5′-CTCAAACTTCCTTGCGTTAGAC-3′) using Primer3Plus [75]. We constructed a reference plasmid containing the target *Hepatozoon* 18 S rDNA fragment (171 bp) amplified from a positive *P. hispanica* individual (JX531917) that we used as amplification standard for the qPCR assay. The PCR product was cloned to a plasmid using the TOPO TA Cloning kit (Invitrogen, California) following the manufacturer's instructions. One Shot TOP10 Competent Cells were used for transformation and we used the Purelink Quick Plasmid Miniprep Kit (Invitrogen, California) to isolate the plasmid (aliquots are available upon request). Successful plasmid integration of the *Hepatozoon* gene fragment was assessed using M13 primers following manufacturer's instructions. Serial 10-fold dilutions containing from 500,000 to 5 copies of plasmid were performed to produce a standard curve for *Hepatozoon* quantification that was included in each qPCR plate. A MyiQ qPCR machine (Applied Biosystems) was used with the following protocol: 95°C 10 min, 95°C 10 s, 63°C 20 s, 72°C 25 s (with melting), 95°C 1 min, 63°C 30 s, melting from 63–95°C by increments of 0.2°C every 10 s. Reactions contained iQ SYBR Green supermix at 0.5x, each primer at 0.5 mM, 2 µl of 10 ng/µl genomic DNA, in a total volume mix of 20 µl. To estimate the number of copies in unknown samples, raw qPCR results were exported using the program iQ5 R&D version 2.1 (Biorad) and the baseline threshold was determined individually for each plate using the algorithm implemented in LinRegPCR [76]. Validation was performed by direct sequencing on a total of 33 qPCR positive individuals. These included qPCR positives displaying a single peak (negatives and positives for *Hepatozoon* from microscopy and PCR) and all those displaying mixed infection peaks. These sequences have been deposited in GenBank under accession numbers KJ189434-KJ189463. For this experiment, samples with all threshold cycles (CTs) higher than 35 were considered negative because repeatability decreased significantly after cycle 35, with most replicates differing by more than one Ct. Mixed infection intensity estimates (*n* = 6) were discarded from subsequent analyses.

### Statistical analyses

Normality tests were performed using Shapiro-Wilk and homogeneity of variances tested using Levene's non-parametric test. To approach normality, infection estimates obtained from qPCR (*Hepatozoon* copy number) were log-transformed using the formula *log (x+1)*. Fitness of the models was tested by Normal Q-Q plots of the Pearson residuals from the model analysis.

#### Between methods' comparisons

We first investigated the correlation between *Hepatozoon* intensity levels detected by microscopy and qPCR using the Spearman correlation coefficient (*n* = 66, out of 72 blood smears after excluding 6 mixed infections). We used McNemar's test to compare sensitivity of the three detection methods (*n* = 87) and the four extraction protocols (*n* = 47) in estimating *Hepatozoon* prevalence. Then, to compare *Hepatozoon* intensity among biological sources and extraction protocols (*n* = 41, out of the subset of 47 samples after excluding 6 mixed infections) we fitted a Generalized Linear Mixed Model (GZLMM), as recommended for unbalanced data with repeated measures (random effects) [77], with normal distribution and log link function. The full model included copy number as the target variable, the biological source, extraction protocol and their interaction as fixed factors, and subject individuals as random effect. Pairwise LSD mean contrasts for each factor were included in the model.

#### Between hosts' comparisons

Previous studies have found various associations between infection parameters and body size [43], [51], and therefore prior to building the models we first examined variation in body size between host species and sexes using ANOVA with Type I sums of squares (SS). For this analysis, body size was the response variable and host species and sexes (and their interaction) were treated as fixed factors. We then tested for inter- and intra-specific associations between body size and intensity of infection using the Spearman correlation coefficient. A positive correlation was found and body size was used as a covariate in all subsequent analyses. Then, using qPCR estimates as a proxy, we investigated differences in infection parameters between the two *Podarcis* host species and between sexes within each species. Prevalence of *Hepatozoon* infection between host species, between sexes within each species and between the same sexes of both species (*n* = 87) was compared using a Chi-square test (http://www.quantpsy.org/chisq/chisq.htm). For intensity comparisons between host species and sexes (*n* = 81, out of 87 samples after excluding 6 mixed infections), while accounting for the effects of body size, we used a Generalized Linear Model (GZLM) with normal distribution and log link function using Type I SS. The full model included body size, host species and sexes (and their interactions) as fixed factors.

All statistical analyses were conducted in IBM SPSS Statistics 21, except for McNemar tests that were conducted in R (R version 3.0.2, R Development Core Team).

## Results

### qPCR assay validation

Intensity of *Hepatozoon* infection estimated by microscopy and qPCR were significantly correlated (ρ = 0.893, *P*<0.001), with qPCR being much more sensitive and detecting as few as 5 copies of parasite DNA. Mean threshold cycle (Ct) standard deviation (SD) was 0.16, mean efficiency 81.1% (SD±2.6) and mean R^2^ = 0.991 (SD±0.008) (Fig. S2). Mean SD values for intra-assay repeatability for sample replicates was 0.198 (SD±0.147) and 0.192 for plasmid dilution replicates (SD±0.166). Melting Curve Analysis of plasmid dilutions displayed a single peak at 81.4°C and amplification charts were S-shaped. Sequences retrieved from qPCR amplifications were BLASTed on GenBank. Those samples displaying a single melting peak curve all matched *Hepatozoon* spp. sequences, while the ones displaying two distinct peaks (range from 82.6–83.8°C) presented a mixed electropherogram in the sequencing analyses. These mixed infections detected by qPCR matched those detected by conventional PCR sequencing.

### Parasite diversity and identification

**Figure 2:**
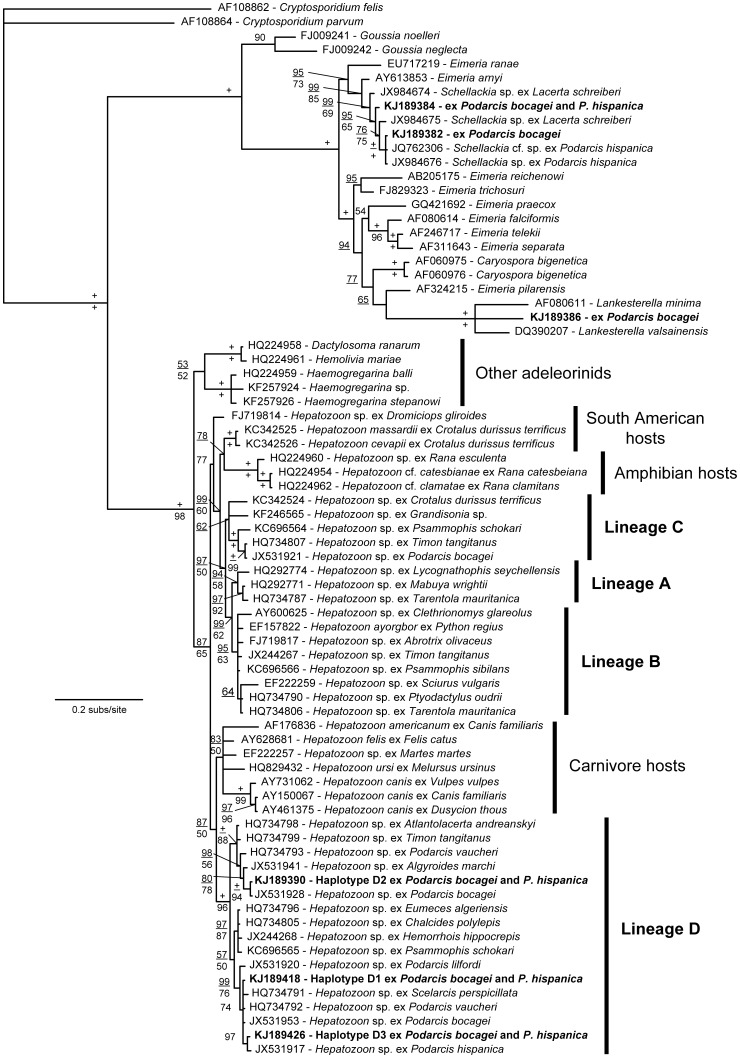
Phylogenetic relationships for the 18 S rRNA gene of the hemoparasites analyzed in this study. Haplotypes retrieved from this study are in bold. Letters refer to lineages found previously in North African lizards [122].

A total of 56 sequences were obtained from PCR sequencing with the Hep primers. Of these, 51 BLASTed with previously published GenBank sequences of *Hepatozoon* spp. (Apicomplexa: Adeleorina), 4 with *Schellackia* spp. and 1 with *Lankesterella* spp. (Apicomplexa: Eimeriorina). These were considered positive for PCR prevalence (Table 1). In mixed infections, these conventional PCR primers preferentially amplified Eimeriorina parasites so they resulted in single electropherograms by sequencing analysis. The 47 *Hepatozoon* sequences (4 were discarded from further analyses due to poor quality) resulted in 3 unique haplotypes (and heterozygous individuals for these haplotypes, see Table S1) that differed in 15 polymorphic segregating sites. These haplotypes clustered within a previously identified *Hepatozoon* lineage obtained from Western Mediterranean and North African reptiles (Fig. 2). The melting curve analysis (MCA) was able to differentiate between different Apicomplexa genera in mixed infections (see above). But we did not find differences in melting temperatures between the three closely related *Hepatozoon* haplotypes, which are identical for the targeted fragment in the qPCR assay (Fig. S1). Two haplotypes of *Schellackia* sp. were obtained, one haplotype from a single individual of *P. bocagei* was identical to *Schellackia* sp. from *P. hispanica* (JQ762306 and JX984676), and the other haplotype (two sequences from *P. hispanica* and one from *P. bocagei*) was closely related with two haplotypes from *Lacerta schreiberi* (JX984674 and JX984675) (Fig. 2). Finally, the single *Lankesterella* sp. sequence obtained in this study is related with *Lankesterella minima* from the American bullfrog *Lithobates (Rana) catesbeianus* (AF080611) and *Lankesterella valsainensis* from the blue tit *Parus caeruleus* (DQ390207) (Fig. 2).

### Methodological comparison

We first compared the accuracy and sensitivity of different detection methods for estimating *Hepatozoon* prevalence, including microscopy, PCR and qPCR (Table 1). The qPCR assay gave the highest prevalence estimates and differed significantly from microscopy (*n* = 72, McNemar Chi^2^ = 17.053, df = 1, *P*<0.001) and PCR (*n* = 87, McNemar Chi^2^ = 11.529, df = 1, *P*<0.001). Microscopy and PCR also differed significantly (*n* = 72, McNemar Chi^2^ = 4.167, df = 1, *P* = 0.041). Among molecular methods: 23% (16/69) of qPCR *Hepatozoon* positives and 75% (3/4) of qPCR Eimeriorina positives were not detected by PCR, while the contrary was found only for 2% (1/51) *Hepatozoon* and 20% (1/5) Eimeriorinid positives (Table 1 and 2). *Hepatozoon* negatives for PCR and qPCR (considering only blood and the kit extraction protocol) were also negative by microscopy, but two Eimeriorina positives by microscopy were not detected by PCR (Table 2).

**Table 2:** False negatives for each of the three detection methods compared in this study.

	False negatives (*Hepatozoon*/Eimeriorina)		
	Microscopy	PCR	qPCR (BK)
Microscopy (*n* = 72)	-	5/2	19/1
PCR (*n* = 87)	0/2	-	16/3
qPCR (BK) (*n* = 87)	0/0	1†/1	-

† Ct was higher than 35 and differed by more than 1 Ct in two trials so was considered negative (see Materials and Methods).

**Figure 3:**
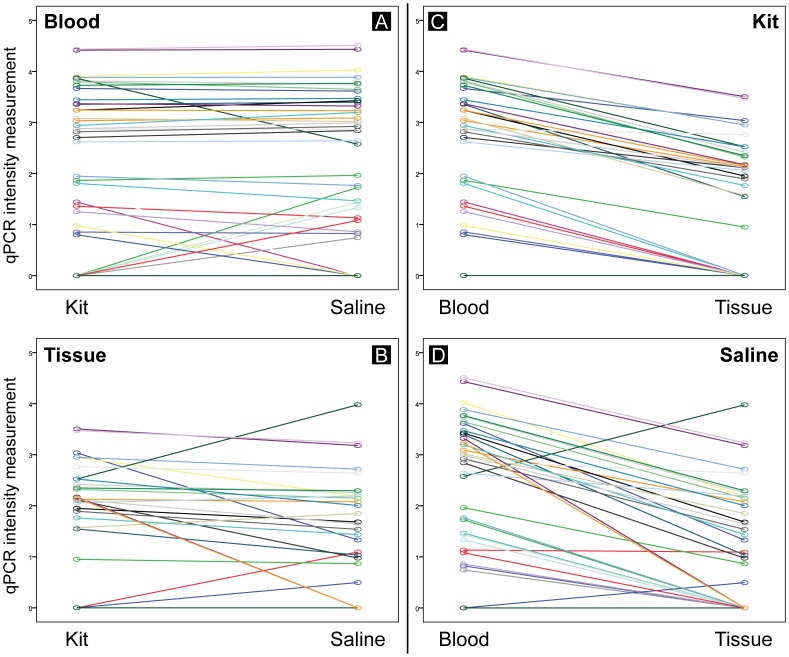
Comparison of the performance of various methods in estimating Hemogregarine infection intensity. The two biological sources: blood (A) and tissue (B), and DNA extraction protocols: kit (C) and saline (D). Each line represents the logged *Hepatozoon* intensity measurement from qPCR for an individual extraction (*n* = 41, out of the subset of 47 samples after excluding 6 mixed infections).

Then, based on the qPCR estimates, we compared the performance of two biological sources (blood and tissue) and two extraction protocols (kit and saline) in estimating *Hepatozoon* infection for a subset of 47 individuals (Table 3). Prevalence differed significantly for the type of source (McNemar Chi^2^ = 10.083, df = 1, *P*<0.001 for kit, and McNemar Chi^2^ = 7.692, df = 1, *P* = 0.003 for saline), but not for the type of extraction protocol (McNemar Chi^2^ = 0, df = 1, *P* = 1.000 for blood and for tissue). In terms of *Hepatozoon* intensity of infection, the GZLMM analysis showed that *Hepatozoon* intensity differed significantly between sources (F = 155.096, df1 = 1, df2 = 160, *P*<0.001) (Fig. 3C and 3D) and between extraction protocols (F = 4.526, df1 = 1, df2 = 160, *P* = 0.035), but their interaction was marginally significant (F = 3.736, df1 = 1, df2 = 160, P = 0.055). Pairwise comparisons further show that the two tissue extractions differ significantly (F = 4,980, df1 = 1, df2 = 160, *P* = 0.027) (Fig. 3B), but not for the two blood extractions (F = 0.046, df1 = 1, df2 = 160, *P* = 0.830) (Fig. 3A), which shows that the previous differences between extraction protocols for the GZLM analysis are due to differences observed between tissue extractions. That is, for tissue the saline method provides slightly lower *Hepatozoon* intensity estimates than the kit (Table 3). Overall, however, both protocol extractions result in proportional estimates of *Hepatozoon* intensity (Fig. 3A and 3B) and provide similar number of false negatives (Table 3). In addition, for the same extraction protocol, tissue provided proportionally lower intensity estimates compared to blood (Fig. 3C and 3D). Finally, blood extractions performed similarly at detecting overall prevalence but differed for samples with extremely low parasitemia levels (Fig. 3A and Table 3).

**Table 3:** Prevalence and mean intensity levels of *Hepatozoon* and prevalence of Eimeriorina parasites using different biological sources (blood and tissue) and extraction protocols (kit and saline) on a subset of samples tested with qPCR.

		*Hepatozoon*		Eimeriorina	False negatives (*Hepatozoon*/Eimeriorina)			
Source	Extraction	Prevalence	Mean Intensity (log(copy number))	Eimeriorina Prevalence	Blood Kit	Blood Saline	Tissue Kit	Tissue Saline
Blood	Kit	38 (81%)	2.17±0.23	4 (9%)	-	5/1	0/2	0/1
	Saline	37 (79%)	2.17±0.23	3 (6%)	6/2	-	0/1	1/0
Tissue	Kit	26 (55%)	1.30±0.19	2 (4%)	12/4	10/2	-	2/0
	Saline	26 (55%)	1.09±0.18	1 (2%)	12/4	11/2	2/1	-
		*n* = 47	*n* = 41	*n* = 47				

### Between-host differences in infection patterns

**Figure 4:**
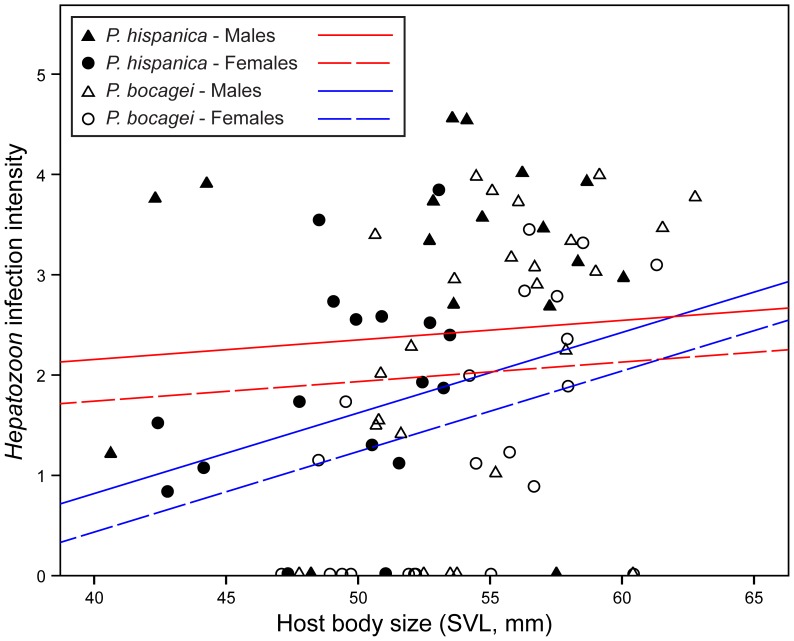
Relationship between *Hepatozoon* intensity and host body size (SVL). Different host species are represented by colours and sexes by shapes. Fit lines were calculated from the parameter estimates of a regression model only considering the significant factors (intensity∼body size+host species+sex+host species*body size).

ANOVA analyses showed significant differences in body size between host species (F = 11.991, df = 1, *P* = 0.001), *P. bocagei* being bigger, and sexes (F = 5.023, df = 1, *P* = 0.028), males being bigger in size. Given that qPCR and blood-kit DNA extraction provided good estimates of infection parameters for the methodological subset comparison, we used this protocol for investigating the relationship between host-factors and infection patterns. Prevalence was higher in *P. hispanica* females (89%) than in *P. bocagei* females (59%) (X^2^ = 4.409, df = 1, *P* = 0.036), but there were no significant differences between species (X^2^ = 3.434, df = 1, *P* = 0.064), or sexes (*P. bocagei*: X^2^ = 3.519, df = 1, *P* = 0.061; *P. hispanica*: X^2^ = 0, df = 1, *P* = 1) (see Table 1). In terms of intensity of infection, intensity was significantly related with body size for both host species (*P. bocagei*, ρ = 0.521, *P*<0.001, and *P. hispanica*, ρ = 0.417, *P* = 0.014) (Fig. 4). The GZLM analysis showed a significant effect of host species (Wald X^2^ = 8.407, df = 1, *P* = 0.004) and sexes (Wald X^2^ = 7.208, df = 1, *P* = 0.007), on *Hepatozoon* intensity levels, and confirmed a significant effect of the covariate factor body size (Wald X^2^ = 4.254, df = 1, *P* = 0.039). The interaction between body size and host species was also significant (Wald X^2^ = 4.571, df = 1, *P* = 0.033), which indicates that the effect of host species on the intensity of infection is body-size dependent (Fig. 4).

## Discussion

### Methodological recommendations

In this study we compared the performance of various detection methods and DNA extraction protocols, and evaluated how the detection method can constrain the estimation of biological relevant infection parameters. First, by comparing various methodological approaches, we found that qPCR was the most accurate and sensitive method for estimating *Hepatozoon* prevalence and intensity, especially in cases of low intensity levels, which is concordant with previous studies conducted on *Plasmodium* [21], [22]. To our knowledge, this is the first quantitative PCR assay of *Hepatozoon* parasites of reptiles, with the few qPCR studies on *Hepatozoon* focusing on carnivore hepatozoonosis [17], [18], [20], [54]. Second, by comparing various extraction protocols on standardized DNA samples, we show that blood samples provide the best estimates of *Hepatozoon* prevalence and intensity levels, regardless of the extraction protocol, and should therefore be the preferred collection method during fieldwork when possible. Our results also show that the traditional saline DNA extraction protocol can be used as an effective low-cost alternative to the tested commercial DNA extraction kit, since it provides similar estimations of hemoparasite infections. This result is in agreement with previous parasitological studies using alternative protocols to commercial kits [50], [78]–[80]. At low intensity levels, protocols had different sensitivities, which pinpoints that performing a preliminary methodological comparison prior to the commencement of the study can be a cost/time effective strategy. Finally, the performance of tissue samples was poorer than blood, with higher number of false negatives for an equivalent DNA concentration (i.e. 10 ng/µl of total DNA). However, tissues still represent a valuable biological source in parasitological studies because voucher specimens and tail-tip tissue have been traditionally collected in reptile studies, and can provide information about past infections and the evolution of specific pathogens [81]. For each DNA extraction protocol, *Hepatozoon* infection intensity from tissue was proportionally lower than blood, probably because a piece of tail-tip muscle contains less DNA, than a blood drop stored in Whatman filter paper. It is possible that increasing the DNA concentration of tissue extractions used in qPCR would yield similar results to blood, but this was not tested in this study. The same applies to tissue extracted with kit and tissue extracted with the saline protocol, the latter providing lower overall estimates of *Hepatozoon* infection. Thus, we recommend the use of higher DNA concentrations when using tissue samples. Our results highlight the need of comparing multiple methods in a case-base manner as a preliminary step, in order to minimize imperfect detection that can ultimately lead to erroneous ecological and epidemiological inferences [14], [16], [82].

In this study we found mixed infection of *Hepatozoon* (Adeleorina) with two genera of Eimeriorinid parasites (*Schellackia* and *Lankesterella*). These parasites were detected by the three detection methods (microscopy, PCR and qPCR), but with different sensitivities. Conventional PCR using the Hep primers only allow the amplification of either hemogregarines or other apicomplexans, and preferentially amplify non-hemogregarine parasites when present in mixed infections [57]. So this method requires at least one additional PCR run using hemogregarine-specific primers (e.g. HEMO [83]). If mixed infections are suspected, we recommend complementing the qPCR approach with conventional PCR that allows newly discovered parasite lineages to be set in a phylogenetic framework. On the other hand, microscopy and qPCR allow the simultaneous detection and differentiation of mixed infections, but failed to detect a few of the *Schellackia* positives detected by PCR. The false Eimeriorina negative of the qPCR approach could be due to handling errors (e.g. pipetting) or, most likely, because of the preferential binding of the qPCR primers towards *Hepatozoon*. Despite the qPCR melting curve analysis allowed us to differentiate between parasite genera, specificity was not high enough to discriminate at lower levels, i.e. between closely related *Hepatozoon* haplotypes. This result is not unexpected, as the aim of this study was to simultaneously detect and quantify various hemogregarine parasites in reptile samples with high accuracy and sensitivity, so the primer set used herein targets an invariable region of the 18 S rRNA gene (see Fig. S1). Thus, future studies aimed at discriminating between and within closely related hemogregarine species may need to refine the proposed qPCR approach by targeting a more variable region or a faster-evolving gene [84]. But in order to do so, a more comprehensive characterization on hemogregarine diversity and taxonomy in multiple hosts and across populations is needed. Eimeriorina infections estimates agree with previous studies that also report low prevalence of these parasites in *P. hispanica* [56], [57]. This finding is relevant since these coccidian parasites are poorly studied and taxonomy is controversial in some taxa [85], [86]. The sequences obtained here resemble *Schellackia* and *Lankesterella* parasites (Apicomplexa: Lankesterellidae). The fact that they are present in both *Podarcis* species at low prevalence and low intensities may be an indication of the opportunistic nature of the interaction, and/or the fact that this parasite may have detrimental effects on hosts. Co-infection implications in wildlife hosts remain poorly investigated but interaction among parasite heterospecifics in mixed infections is often asymmetrical [3] and the effects of their coexistence may be more detrimental than the additive effects of single infections, resulting in stronger selective pressures on hosts [8], [9]. More sensitive and accurate approaches such as the qPCR assay developed in this study, which simultaneously detects hemogregarine and eimeriorinid parasites, can allow the investigation of the occurrence of mixed infections in natural populations in a systematic and more standardized way.

### Between-host differences in infection patterns

Based on qPCR estimates (from blood-kit DNA extraction) we investigated *Hepatozoon* infection parameters in two *Podarcis* sympatric species. Our results show high levels of prevalence with no significant differences between host species and sexes within each species. Prevalence can be determined by the rate at which parasites encounter suitable hosts [9], environmental factors [87] and by the distribution of suitable vectors [88]. Thus, the observed pattern may arise because these hosts' species share the same habitat and are exposed to the same parasites and vectors, both being suitable hosts for hemogregarine parasites. This finding differs however with results from a previous study showing differences in prevalence in these two host species from different locations in the Iberian Peninsula [50]. These contrasting patterns may be the result of the differences between method detection accuracy, as shown in this study, or due to the influence of temporal, ecological, evolutionary and behavioural factors in hemoparasite infection [6], [44]. Interestingly, we found three haplotypes and heterozygous individuals, consistent with what has been previously reported by other studies [50]. The importance of this variation should be further studied as the co-occurrence of various parasite species and lineages within a host may contribute increasing parasite and/or host diversity and speciation [89] and parasite clonal diversity can maintain high parasite diversity in host populations [90]. Clearly, integrative studies that involve all these variables are needed in wild reptiles.

Regarding *Hepatozoon* intensity of infection, we found that it was positively associated with body size within each host species, which seems to be a common pattern among short-lived reptile species (e.g. *Iberolacerta monticola* [43], *P. lilfordi* [51] and *Lacerta viridis* [91]). Body size has been used as an estimator of age in reptiles [92], [93], with older individuals of the same species usually being larger in size. It is assumed that intensity of infection may increase with longevity due to more encounters with parasites, more time to develop infections and less immunocompetence in older animals [37], [42], [94]. However, this pattern is not linear among hemogregarine infections, especially when considering long-lived reptile species. Studies conducted on snakes [35], [36], [95] and tuataras [96] have found an inverse association to that reported here, with larger individuals having lower intensities. These contrasting infestation patterns may be related to differences in the pathogenesis of hemogregarine infections between short- and long-lived reptiles. In short-lived species, such as lizards, hemogregarine infections are often regarded as non-detrimental to host condition [97], while in long-lived species hemogregarine infection appears linked to detrimental effects on life-history traits, including growth rate, juvenile survival and female reproductive output [35]. These contrasting effects may be due to the continued parasite acquisition through time in larger, and older, individuals, in the case of non-detrimental infections [26], while in detrimental infections only those individuals that manage to reduce or clear infection can have longer lifespans and reach larger sizes [9], [35]. Given the strong relationship of host longevity and body size on parasite infection in reptiles in nature, it would be interesting to investigate hemogregarine infection patterns in species with intermediate size and longevity to determine if such an intermediate parasite infection pattern is found.

When comparing intensity levels between the two species, the smaller sized species (*P. hispanica*) harboured significantly higher intensity levels of *Hepatozoon* parasites. The association between body size and intensity of infection differed for the two species, being more pronounced in *P. bocagei*, for which a lower proportion of smaller animals and a higher proportion of bigger animals, are infected. Also, males had higher intensities than females. Several factors may explain these patterns including: host immunocompetence and susceptibility to infection [41], [98], [99], parasite specialization to both the intermediate [44] and definitive hosts [100]–[102], host microhabitat preference [88], abundance of suitable vectors [101], [103], [104], and behaviour heterogeneity of host species and sexes [9], [40], [105]. In our system, the smaller-sized species, *P. hispanica*, and males within each species, can be more susceptible to infection and/or can differ immunologically/hormonally, and thus harbour higher hemogregarine intensity levels. Differences in leukocyte counts have been reported between *Podarcis* species [106], which can result in differential immune responses. Associations between individual- and species- specific immune responses and parasitemia levels for various blood parasite groups have been widely reported in birds [107]–[110], and, to a lesser extent, in reptiles [95], [111]. Likewise, a male-biased pattern is commonly observed in hemoparasite infections, which is often attributed to hormonal and immunocompetence differences between sexes [112]–[114]. Future studies should now look for an association between immunological variables and hemogregarine infection in sympatric species.

Hemoparasite infection parameters can vary temporally and spatially, which is especially important in complex parasite systems that have heteroxenous lifecycles and are vector-borne, such as *Hepatozoon*. For instance, a cross-sectional study of two malaria parasite species has shown parasite-specific variation in the spatial patterns of disease risk in two closely related sympatric bird species, with consistency between years [115]. Seasonal variations (or environmental variations due to climate change [116]) can not only affect vector abundance [117] and influence rates of parasite development in these vectors [118], but also influence vertebrate host behaviour [119] and host immune condition [120], [121]. So studies on various populations and/or across different seasons and years should be conducted in these sympatric species, provided that this system presents a good host-parasite interaction assessment for comparing differences between species and sexes. Finally, a fundamental question on the biology of these parasites that remains, is to identify and compare host-specificity and competence of the definitive invertebrate host(s) for reptile hemogregarines which is fundamental to better understand the evolutionary ecology and transmission dynamics of these parasites.

## Conclusion

Here, we present a qPCR assay to detect and quantify hemogregarine hemoparasites in reptiles, which can also differentiate mixed infections by different apicomplexan genera. We then tested the performance of the qPCR approach in natural settings and compared infection patterns in a pair of lizard species. Our study illustrates that closely related sympatric hosts, for which factors like behaviour, morphology and habitat preferences are well-known, represent a good model for studying among-host variation in infection. Future studies are needed to assess the influence of other intrinsic factors, such as physiological and immunological traits, as well as to investigate the evolutionary and ecological significance of mixed infections. Overall, this study illustrates the importance of using accurate detection and quantification methods for estimating infection parameters, and the need of considering methodological aspects when comparing parasitological data within and between studies. We also advocate that, due to differences in performance between detection methodologies and extraction protocols, preliminary studies should be carried out prior to choosing the most appropriate approach in a case-study basis to avoid erroneous inferences.

## Supporting Information

Figure S1 Sequence alignment of the 18 S rRNA gene fragment targeted by the qPCR assay. Arrows indicate nucleotide positions that differ between major *Hepatozoon* lineages (see Fig. 2).
(TIF)

Figure S2 Standard curve obtained from a six 10-fold serial dilutions of the plasmid containing the 18 S rRNA gene. Numbers correspond to dilutions from 1 (500,000 copies) to 6 (5 copies). Dashed lines indicate the number of copies for an unknown sample determined based on the starting Threshold cycle (Ct).
(TIF)

Table S1 Number of haplotypes and heterozygous individuals found in this study. The symbol + indicates when the first haplotype peaks are higher, while & indicates when both haplotype peaks are approximately of the same height.
(DOC)
