# Supplementary figures and images for: A Comparison of Multiple Methods for Estimating Parasitemia of Hemogregarine Hemoparasites (Apicomplexa: Adeleorina) and Its Application for Studying Infection in Natural Populations
Source: PLoS One. 2014 Apr 17;9(4):e95010. doi: 10.1371/journal.pone.0095010 (PMC3990604; doi:10.1371/journal.pone.0095010)

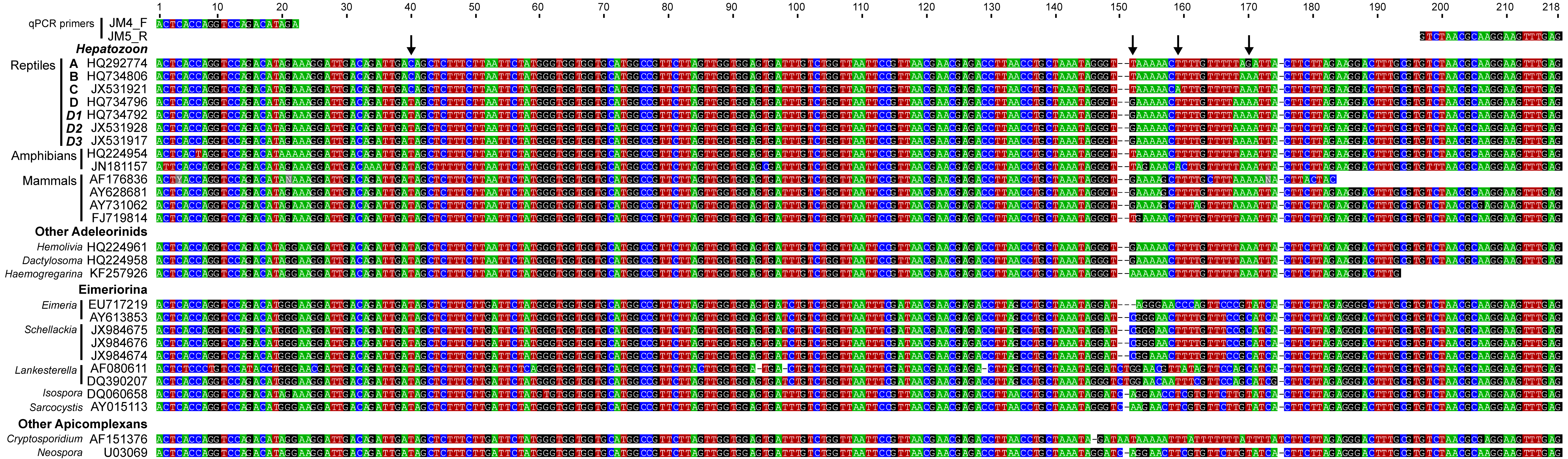

Supplement: Figure S1 — Sequence alignment of the 18 S rRNA gene fragment targeted by the qPCR assay. Arrows indicate nucleotide positions that differ between major Hepatozoon lineages (see Fig. 2). (TIF) [file pone.0095010.s001.tif]

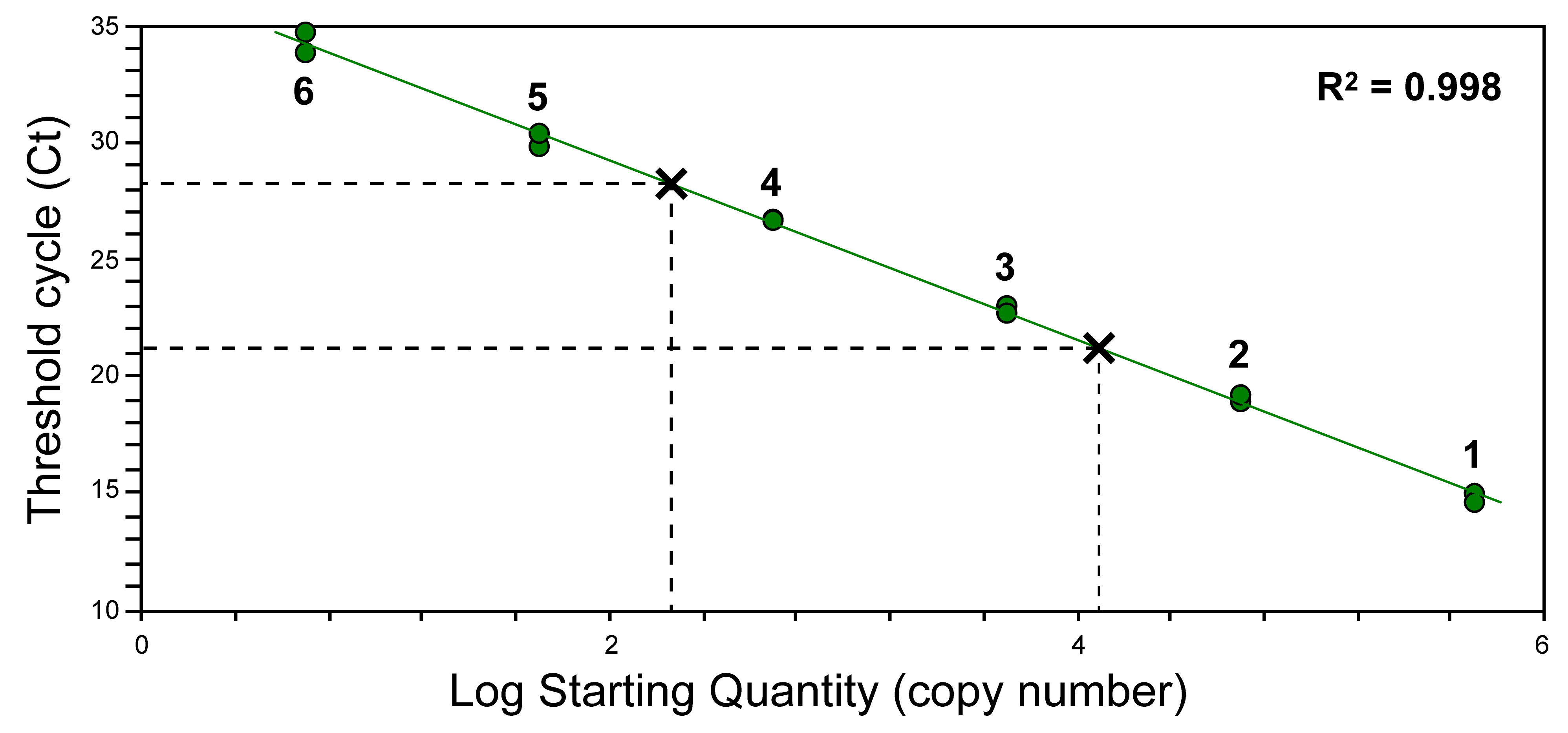

Supplement: Figure S2 — Standard curve obtained from a six 10-fold serial dilutions of the plasmid containing the 18 S rRNA gene. Numbers correspond to dilutions from 1 (500,000 copies) to 6 (5 copies). Dashed lines indicate the number of copies for an unknown sample determined based on the starting Threshold cycle (Ct). (TIF) [file pone.0095010.s002.tif]
