# Supplementary material for: A Comparison of Multiple Methods for Estimating Parasitemia of Hemogregarine Hemoparasites (Apicomplexa: Adeleorina) and Its Application for Studying Infection in Natural Populations
Source: PLoS One. 2014 Apr 17;9(4):e95010. doi: 10.1371/journal.pone.0095010 (PMC3990604; doi:10.1371/journal.pone.0095010)
Supplement: Table S1 — Number of haplotypes and heterozygous individuals found in this study. The symbol + indicates when the first haplotype peaks are higher, while & indicates when both haplotype peaks are approximately of the same height. (DOC) [file pone.0095010.s003.doc]

**Supporting Information**

Table S1. Number of haplotypes and heterozygous individuals found in this study. + indicates when the first haplotype peaks are higher. & indicates when both haplotype peaks are approximately of the same height.

|  |  | **Haplotypes** | | | **Heterozygotes** | | |
| --- | --- | --- | --- | --- | --- | --- | --- |
| **Host species** | **Sex** | **D1** | **D2** | **D3** | **D1+D2** | **D2+D1** | **D1&D2** |
| *Podarcis bocagei* | Female | 2 | 2 | 1 | 3 | 2 | 0 |
|  | Male | 2 | 6 | 2 | 3 | 0 | 2 |
|  |  | 4 | 8 | 3 | 6 | 2 | 2 |
| *Podarcis hispanica* | Female | 2 | 5 | 1 | 0 | 0 | 2 |
|  | Male | 8 | 1 | 1 | 0 | 1 | 1 |
|  |  | 10 | 6 | 2 | 0 | 1 | 3 |
|  |  | 14 | 14 | 5 | 6 | 3 | 5 |
